# COVID-19 disrupted patterns of cause-specific mortality in Switzerland

**DOI:** 10.3389/ijph.2026.1609856

**Published:** 2026-06-23

**Authors:** Anthony Hauser, Moritz Wagner, Anna Fesser, Karim Abawi, Anne Laube, Garyfallos Konstantinoudis, Julien Riou

**Affiliations:** 1 Department of Epidemiology and Health Systems, Unisanté, University Center for Primary Care and Public Health and University of Lausanne, Lausanne, Switzerland; 2 Communicable Diseases Division, Federal Office of Public Health, Bern, Switzerland; 3 Federal Statistical Office, Neuchâtel, Switzerland; 4 Grantham Institute for Climate Change and the Environment, Imperial College London, London, United Kingdom

**Keywords:** Bayesian statistics, cause-of-death statistics, cause-specific mortality, COVID-19, multivariate models

## Abstract

**Objectives:**

To understand disruptions in mortality patterns during the COVID-19 pandemic beyond deaths directly attributed to SARS-CoV-2.

**Methods:**

We analysed Swiss weekly deaths (2011–2021) by age, sex, region, and nine cause groups. For each age group, a Bayesian multivariate Poisson model was fitted to 2011–2019 data, accounting for seasonality, long-term trends, and cross-cause dependence. Comparing predictions with 2020–2021 observations revealed age- and cause-specific excesses.

**Results:**

Cardiovascular deaths peaked during the autumn 2020 wave, while no cause showed sustained excess across 2020–2021. In individuals aged ≥80 years, non-COVID respiratory (−25%) and mental/neurological (−12%) deaths declined, largely offset by COVID-19 deaths, leaving a modest +4.5% all-cause excess. Cardiovascular and mental/neurological excesses correlated with COVID-19 and other respiratory excesses, reflecting strong pre-pandemic cross-cause correlations ( ρ>0.80 ).

**Conclusion:**

These patterns likely reflect three overlapping mechanisms: reduced circulation of non-COVID-19 respiratory pathogens, unrecognized COVID-19 deaths (especially cardiovascular), and mortality displacement, pointing to possible underestimation of the true respiratory burden.

## Introduction

The COVID-19 pandemic has had a profound impact on mortality patterns worldwide. While much attention has focused on deaths directly attributed to SARS-CoV-2 infection, the total mortality burden linked to the pandemic extends well beyond these immediate effects, reflecting multiple interrelated disruptions to mortality patterns occurring over the short, medium and long term. First, non-pharmaceutical control measures considerably reduced deaths from other respiratory diseases by limiting the circulation of seasonal viruses [1]. Second, changes in mobility and social behavior also affected deaths from external causes, with fewer accidents due to reduced mobility and a possible influence of psychosocial stress on suicide risk [2]. Third, in some countries, healthcare disruptions delayed diagnoses and treatment, particularly for cancer, potentially increasing mortality from certain chronic illnesses [3]. Fourth, SARS-CoV-2 infection itself may have contributed to deaths attributed to other causes, especially from cardiovascular causes, either through undiagnosed infection at the time of death or long-term complications [4]. Finally, the COVID-19 pandemic has also induced mortality displacement, both over time and across causes, by disproportionately affecting frail individuals (older people or patients with poor prognoses) and thereby altering the risk profile of the surviving population [5]. Studies excluding COVID-19-attributed deaths have shown that mortality from other causes sometimes fell below expected levels, highlighting these indirect effects [6].

Given the limitations of COVID-19-attributed mortality, which is prone to underascertainment and only captures immediate consequences of infection, other metrics have come to the forefront to measure the burden of infection [7]. All-cause excess mortality, defined as the difference between observed deaths and the counterfactual number that would have been expected in the absence of pandemic, is more robust, as it does not rely on cause-of-death classification and captures the total mortality impact. However, as a composite measure, it cannot identify whether apparent increases in some causes are offset by decreases in others. A natural extension is to estimate cause-specific excess mortality, which allows quantifying the contributions of individual causes over time and, when aggregated, could provide more accurate assessment of total excess mortality [8].

Previous studies on cause-specific excess mortality during the COVID-19 pandemic have documented important shifts in mortality patterns, including increases in cardiovascular deaths alongside declines in respiratory and dementia mortality, with substantial heterogeneity across countries [4], [9–12]. Some investigations have also reported temporal and geographic correlations between COVID-19 activity and cause-specific mortality [9, 10, 13]. However, most studies focused on a limited set of causes and on the exceptional circumstances of the pandemic, without capturing the broader interdependence among causes of death. Yet cross-cause correlation is precisely what is needed to disentangle misclassification, mortality displacement, and indirect pathogen effects from one another. In Switzerland, several studies have examined all-cause mortality and described changes in cause-specific mortality during the pandemic [6, 14, 15], and the Federal Statistical Office has documented shifts in the relative distributions of deaths across cause categories over 2020–2022 [16]. Most recently, Durán et al. found lower estimates of excess mortality from chronic diseases when using underlying rather than multiple causes of death during the COVID-19 wave, supporting the view that COVID-19 may in some cases have displaced chronic conditions as the underlying cause reported on death certificates [17]. Across all these studies, however, mortality series have been analysed separately by cause, and no existing work has jointly modelled their temporal dynamics throughout the pandemic.

Switzerland maintains high-quality mortality statistics through a thorough process of reviewing each death certificate, yielding rich information on underlying, immediate, and concomitant causes suitable for detailed epidemiological analyses [18]. Leveraging these data, we aimed to estimate cause-specific excess mortality in Switzerland from 2011 to 2021, with a particular focus on cross-cause correlations. We implemented a Bayesian multivariate model that captured seasonal and long-term trends, accounted for population dynamics, and models correlation across causes through a shared latent random effects structure. By examining both the pre-pandemic synchrony in cause-specific mortality and its disruption during the pandemic, we aimed to identify signals of cause-of-death misclassification, mortality displacement, and indirect effects of COVID-19. This approach, by highlighting the interdependence of mortality processes, offers new insight into the mechanisms by which the pandemic and the related control measures have reshaped mortality patterns.

## Methods

### Data

We received individual-level data on cause-specific deaths for the years 2011–2021 from the Federal Statistical Office (FSO), which we aggregated by age group (0–17, 18–39, 40–64, 65–79, 80+), sex, NUTS-2 region, calendar week, and calendar year. The data include information recorded in the death certificate—such as the immediate, underlying, and contributing (concomitant) causes of death, as well as the official underlying cause of death, which is determined by the FSO based on the coding rules of the International Classification of Diseases, 10th Revision (ICD-10) [19]. All causes of death are coded using ICD-10 and were grouped into nine categories for analysis: cardiovascular diseases (Chapter IX), cancers (Chapter II), mental and neurological disorders (Chapters V and VI), respiratory diseases (Chapter X), External causes (Chapter XX), suicide (Chapter XX), infectious and parasitic diseases (Chapter I), other causes (Chapters III, IV, VII, VIII, XI–XIX, XXI, XXII), and COVID-19 (Chapter XXII) (see Supplementary Table S1). We modelled suicide separately to assess potential pandemic-related changes, given early concerns regarding the impact of restrictions on suicide mortality. Yearly population data by sex, NUTS-2 region, and age group for the years 2011–2021 were also provided by the Federal Statistical Office (FSO). We applied linear interpolation to obtain weekly population estimates.

### Model

We modeled the weekly number of deaths by cause for each age group using a Poisson distribution with a multivariate structure under a Bayesian framework, considering only the underlying cause of death. The model accounts for changes in population size N over time via an offset term, and includes fixed effects β and γ for differences in cause-specific mortality risk by region and sex, respectively. Temporal variation was captured by two components: a seasonal trend modeled using a periodic Gaussian Process (GP) with an exponentiated sin-squared kernel $f\left(t\right)$ , and a long-term trend modeled using a GP with an exponentiated quadratic (squared exponential) kernel $g\left(t\right)$ [20], with t representing calendar weeks. To account for the correlation between mortality risks across causes, the model incorporated a multivariate random effect $\epsilon \left(t\right)∼N\left(0,\Sigma \right)$ , where Σ is the covariance matrix. Correlations between causes were modeled using a Cholesky factor with LKJ(1) prior, which imposes no *a priori* preference on the strength or direction of the correlations.

The model is specified as follows:

$$y_{irs}\left(t\right)∼\text{Poisson}\left(\mu_{irs}\left(t\right)\right)$$

with

$$\log\;\mu_{irs}\left(t\right)=\alpha_{i}+\beta_{ir}+\gamma_{is}+f_{i}\left(t\right)+g_{i}\left(t\right)+\epsilon_{i}\left(t\right)+\log N_{irst},$$

where $y_{irs}\left(t\right)$ denotes the observed number of deaths due to cause i in week t , region r , and sex s , and $\alpha_{i}$ is the baseline log-transformed mortality risk. Note that even though we used a Poisson likelihood, this model accounts for potential overdispersion through its complex random-effect structure.

The model was run independently for each age group and fitted to the weekly number of cause-specific deaths $y_{irs}\left(t\right)$ observed between 1 January 2011 and 31 December 2019. It was then used to estimate the weekly *expected number of deaths* ${\tilde{y}}_{irs}\left(t\right)$ in 2020 and 2021, defined as the *posterior predictive distribution* after removing the random effect $\epsilon \left(t\right)$ from the log mortality risk $\mu_{irs}\left(t\right)$ . We removed $\epsilon \left(t\right)$ because it was designed to capture *excess mortality*, i.e., unexplained or anomalous variation beyond structured trends. Weekly excess mortality by cause, region and sex $E_{irs}\left(t\right)$ was then defined as the difference between observed and expected deaths:

$$E_{irs}\left(t\right):=y_{irs}\left(t\right)-{\tilde{y}}_{irs}\left(t\right).$$

We also reported aggregated estimates of excess mortality (i.e., *all-cause excess mortality*), across regions, across sexes and across time periods. This was simply obtained by adding posterior samples.

### Further analysis

We examined how excess deaths from specific causes were related over time to excess deaths from COVID-19 during 2020–21. To do this, we computed partial Pearson correlations between posterior samples of cause-specific excess deaths and COVID-19 deaths, while controlling for excess deaths from other respiratory diseases. This adjustment aimed to separate the association with COVID-19 from that with other circulating respiratory pathogens. We also explored the effect of time lags, by shifting the time series of cause-specific excess deaths up to 8 weeks forward and backward, to assess whether the strength of association varied depending on timing. We performed the same analysis to assess the relationship between cause-specific excess deaths and excess deaths due to (non-COVID) respiratory diseases, this time adjusting for COVID-19 mortality.

To better understand the observed trends in deaths with cancer as the underlying cause, we conducted an additional analysis including all individuals who died *with* cancer, i.e., where cancer was reported either as the underlying (referred to as *deaths from cancer*) or the immediate cause of death. We computed the number of deaths from cancer to the total number of deaths with cancer over time and examined their distribution by cancer survival rate to assess whether competing risks with COVID-19 might explain some of the observed patterns. We performed all the analyses in R version 4.4.1 and Stan version 2.35.0 [21]. All code is available from https://github.com/anthonyhauser/causes-of-deaths.

## Results

### Distribution of deaths

**FIGURE 1:**
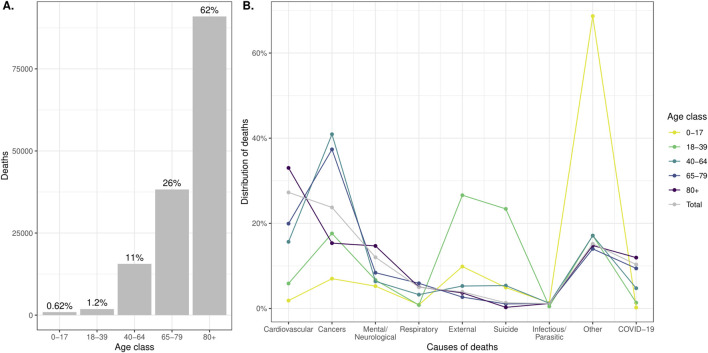
Cause-of-death data in 2020–21. **(A)** Number of deaths by age groups in 2020–21. **(B)** Distribution of the deaths occured in 2020–21 over the nine causes of deaths for each age group (Switzerland, 2011–2021).

In Switzerland, deaths were heavily concentrated among older adults: 88% occurred in individuals aged 65 and over, while fewer than 2% were among people under 40 in 2020–21 (Figure 1A). Cardiovascular diseases were the leading cause of death overall, followed by cancers and mental or neurological disorders, though the distribution varied by age group (Figure 1B). Among individuals aged 80 and above, cardiovascular disease accounted for 33% of all deaths. In contrast, for those aged 40–79, cancer was the predominant cause, responsible for approximately 40% of deaths. The relative impact of COVID-19 was greatest in older age groups, contributing to 9% of deaths among those aged 65%–79% and 12% among those aged 80 and over.

### Prepandemic mortality trends

**FIGURE 2:**
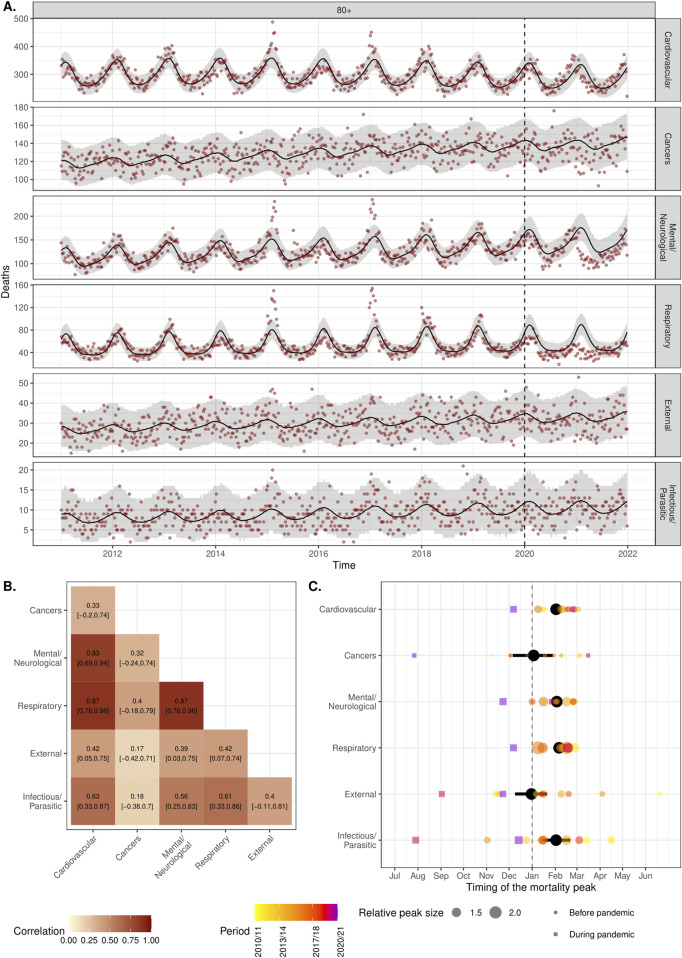
Model fit and cross-cause dependencies for the 80+ age group. **(A)** Model fit. Number of weekly deaths observed in 2011–21 (red points) and model estimates (before 2020) and predictions (2020–21) (black line) with 95% credibility intervals (gray area), for the six main causes of deaths for the 80+ age group. **(B)** Cross-cause correlation matrix of the excess mortality for 80+ age group. **(C)** Timing of the mortality peak. The color points and squares correspond to observed peak for each winter (we considered the period from July to June of the next year as peak usually occured during winter). The black points and lines represent the posterior estimate of the peak with 95% CrI (Switzerland, 2011–2021).

The model captured the seasonal and long-term trends in cause-specific mortality between 2011 and 2019 (Figure 2A for individuals aged 80+, and Supplementary Figures S1–S4 for other age groups), while rare episodes of excess mortality were identified, such as in the winters of 2014/15 and 2016/17. We observed similarities in mortality trends across six different groups of causes, both in the seasonal patterns and in intermittent episodes of excess mortality that often occur synchronously. For the 80+ age group, Figure 2C highlights this synchrony in timing, showing that the expected mean mortality peak (in black) occured consistently across causes between January and February. During the pre-pandemic period (2011–2019), observed peak timings align closely with these expectations. However, during the pandemic years, we observed a marked shift: mortality peaks occur earlier than expected. In addition, the model revealed strong positive correlations in cause-specific excess mortality, mostly among individuals aged 80 and over (Figure 2B). Specifically, excess deaths from respiratory diseases, mental or neurological disorders, and cardiovascular diseases were highly correlated, with pairwise correlations exceeding 0.8.

### COVID-19 mortality

**FIGURE 3:**
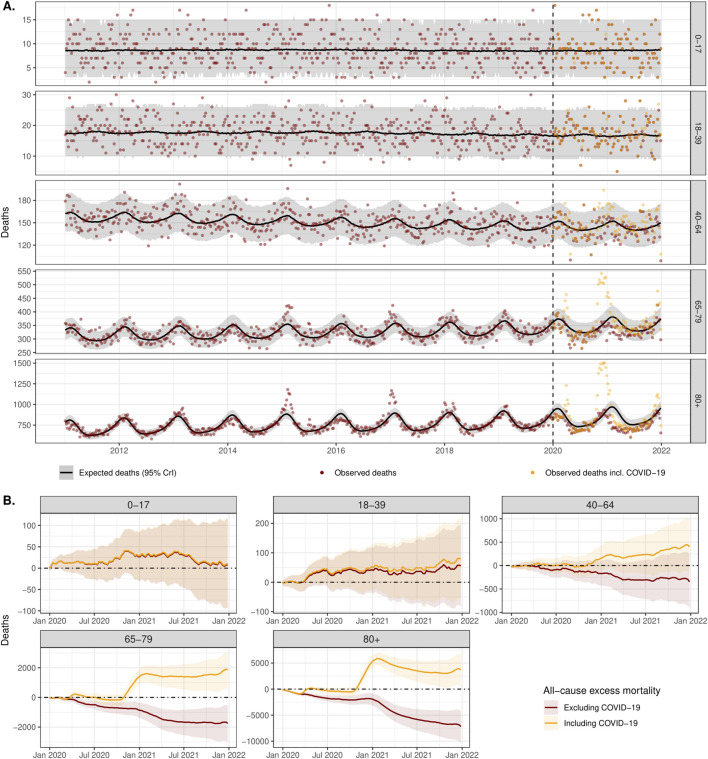
All-cause mortality. **(A)** Number of weekly all-cause deaths (i.e., aggregated over all causes of deaths) for each age group. The red points correspond to the observed mortality without COVID-19 and the yellow ones with COVID-19. Black lines and shaded areas correspond to the model posterior mean (i.e., expected mortality) with 95% credibility intervals. **(B)** Cumulative number of excess deaths with (in yellow) or without (in red) COVID-19 deaths, for each age group (Switzerland, 2011–2021).

The model also captured all-cause mortality between 2011 and 2019, obtained by aggregating the expected cause-specific mortality. Among individuals aged 65 and over, the impact of the first two COVID-19 waves, during spring 2020 and fall/winter 2020–21, clearly exceeded any episodes of excess mortality observed between 2011 and 2019 (Figure 3A). Cumulative excess mortality highlights the substantial increase in deaths during these waves, followed by a gradual mortality deficit in the subsequent weeks (Figure 3B). When excluding deaths attributed to COVID-19, we also observed that mortality from other causes was lower than expected in 2020–21 for people aged 65 and over.

### Cause-specific excess mortality in 2020–21

**FIGURE 4:**
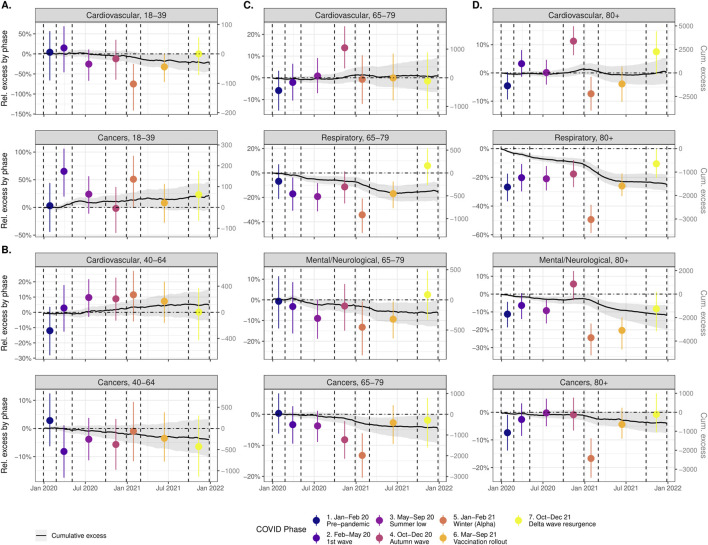
Cause-specific excess mortality by phase. Black lines and gray area correspond to posterior mean and 95% CrI of the cumulative (relative and absolute) excess mortality over 2020–21, respectively. The colored points and vertical lines correspond to posterior mean and 95% credibility intervals of the relative excess mortality by phase, respectively. The phases are delimited by the dashed vertical lines. Panels **(A–D)** represent the different age groups. See Supplementary Figures S5–S9 for the excess mortality by phase for each cause and each age group (Switzerland, 2011–2021).

Among individuals aged below 64, almost all cumulative mortalities remained within expected levels over 2020–21, suggesting no evidence of excess mortality (Figures 4A,B; Supplementary Figures S5–S9). A slight excess in suicides was observed among 0–17 over 2020–21 (16, 95% Credibility Interval (95% CrI) [2–28], +54%), but the small number of observed deaths (43) limits interpretation (Supplementary Figure S5). In contrast, among individuals aged 65–79, a deficit in deaths due to cancers and mental or neurological disorders was observed in 2020–21, with approximately 5% fewer deaths than expected (Figure 4C). The largest decrease occurred during the Alpha wave in January–February 2021. Mortality due to cardiovascular diseases in this group remained within the expected range, although a localized excess was noted during the autumn 2020 wave (+14%).

Similar patterns were seen in the 80+ age group (Figure 4D). A marked decrease in mortality from mental and neurological disorders was observed in 2020–21 (−12%), with levels below expectation in all phases except the autumn 2020 wave, when a slight increase occurred. The most pronounced decrease was during the Alpha wave (−24%). For cancer, the mortality deficit was also concentrated almost entirely during the Alpha wave. Cumulative mortality due to cardiovascular diseases among the 80+ was within expected levels overall, but showed substantial variation across phases. Excess mortality was concentrated during COVID-19 waves, while mortality deficits were more common at the beginning of both 2020 and 2021. Finally, we observed a consistent deficit in deaths due to respiratory diseases (excluding COVID-19) across all age groups, with the magnitude of the deficit increasing with age. Supplementary Figures S5–S9 display cause-specific excess mortality estimates by phases for all causes and all age groups.

To better understand the apparent decrease in cancer-related excess mortality in January–February 2021, we examined deaths among individuals aged 65 and over with a cancer diagnosis in 2020–21. Unlike analyses based solely on the underlying cause of death, this approach also included individuals with cancer who died from other causes. In contrast to deaths *from* cancer (pink), the total number of deaths with cancer (black) did not decline during January–February 2021 and even increased slightly in the 65–79 age group and more markedly in those aged 80 and over (Supplementary Figure S10A). When stratifying deaths with cancer by survival category, we observed that cancers with high survival exhibited the largest increase during this period, particularly among individuals aged 80 and over (Supplementary Figure S10B).

### Correlation with respiratory diseases in 2020–21

**FIGURE 5:**
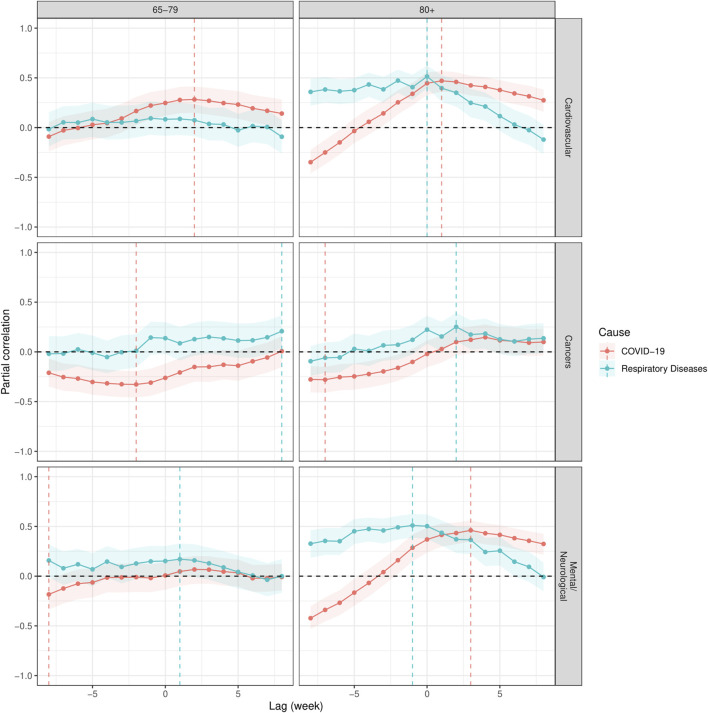
Partial correlation between respiratory excess mortality and other causes. Partial correlation between excess mortality due to either COVID-19 (red) or to other respiratory diseases (blue, tagged as “Respiratory Diseases”) and excess mortality due to cardiovascular diseases, cancers or mental/neurological diseases, for 65–79 and 80+ age groups, considering different lags (in weeks), i.e., different time shifts between changes in excess mortality due to respiratory diseases and changes in excess mortality due to the considered cause. Solid color lines correspond to the posterior mean and shaded colored area to the 95% credibility intervals. The vertical dashed lines indicate the lag with the highest absolute partial correlation. A positive lag corresponds to the situation where excess mortality due to respiratory diseases (either COVID-19 or other respiratory diseases) occurs slightly after excess mortality due to the considered cause (Switzerland, 2011–2021).

We examined the temporal correlation between excess mortality from specific causes and excess mortality due to either COVID-19 or other respiratory diseases during 2020–21. For cardiovascular and mental/neurological diseases, we observed positive partial correlations with COVID-19 mortality, reaching nearly 0.5 among individuals aged 80 and over (Figure 5; Supplementary Figure S11). Similar correlations were also found with other respiratory diseases, reflecting the high correlations inferred by the model for the prepandemic period 2011–2019. The strongest correlations with COVID-19 were seen at lags of 1–3 weeks, meaning that COVID-19-related excess deaths happened slightly after those from cardiovascular or mental/neurological causes. In contrast, we observed negative correlations between cancer and COVID-19 mortality, particularly among those aged 65–79.

## Discussion

### Summary

We modelled cause-specific mortality trends in Switzerland from 2011 to 2019 to assess excess mortality during the COVID-19 pandemic in 2020–2021. Compared with the prepandemic baseline, we identified several disruptions in mortality patterns. Cardiovascular deaths rose temporarily during COVID-19 waves, especially among people aged 65 years and older. By contrast, respiratory-disease mortality declined consistently throughout the pandemic. Among older adults, deaths attributed to mental/neurological diseases and to cancer fell below expectation in 2021. These deficits were more than offset by COVID-19 deaths, resulting in overall all-cause excess mortality. These shifts likely reflect a combination of mechanisms, including mortality displacement, competing risks, and reduced circulation of other pathogens due to public-health measures.

### Potential explanations for the observed patterns: 4 types of disruption

Four broad disruptions could explain the observed patterns. First, under-ascertainment of COVID-19 deaths inflated the counts attributed to other causes. Modelling work suggests that only 72% of COVID-19 deaths were identified as such in Switzerland during 2020–21, with even lower proportions in epidemic peaks [6]. Because infection with SARS-CoV-2 can trigger cardiovascular complications, through inflammation, thrombosis, and direct cardiac injury, deaths with COVID-19 as an unrecognized concomitant cause likely contributed to the cardiovascular excesses seen in autumn 2020 and autumn 2021. This is further supported by the high positive correlations between COVID-19 and cardiovascular excesses.

Second, sequelae of SARS-CoV-2 infection could increase other cause-specific mortality. Cohort studies have documented a higher individual risk of delayed complications, particularly cardiovascular events, months after infection [22, 23]. Population-level analyses show that these delayed effects contributed to sustained excess cardiovascular mortality in 2021 in countries such as the United States [4] and the United Kingdom [24]. Such effects are difficult to detect in the relatively healthy Swiss population and may overlap with other disruptions—such as misclassified COVID-19 deaths or mortality displacement—complicating interpretations.

Third, mortality displacement, i.e., when deaths occur earlier than they would have in the absence of the COVID-19 pandemic, partly explains the deficit in mortality observed during the first part of 2021. The clearest signal appears in mental and neurological disorders: among people aged 65 and over, deaths remained below baseline for several weeks after the autumn 2020 and winter 2020–21 COVID-19 waves. Nordic countries have seen a similar “harvesting” pattern: some individuals with dementia died during COVID-19 waves rather than later, leaving fewer dementia deaths afterward [12]. While displacement can persist for months in slowly progressing disorders such as dementia, its impact may appear almost immediately when remaining life expectancy is short, as in advanced cancers. Using multiple cause of death data, we found that the observed decline in cancer deaths at the end of 2020 was offset by an increase in cancer patients dying from other causes, mainly COVID-19, suggesting displacement from cancer to COVID-19 due to competing risks. This interpretation was supported by the analysis stratifying cancers by survival risk, which showed lower increase in mortality among individuals with low-survival cancers, where the risks of dying from cancer or COVID-19 directly compete. Such results have also been reproduced theoretically through simulation-based modelling work [25]. Finally, the observed negative correlation between cancer-related excess mortality and COVID-19 deaths, which was also reported spatially across US states, further supports this interpretation [13].

Finally, pandemic response, both mandated restrictions and voluntary behavior changes, affected mortality in various ways. Non-pharmaceutical interventions (NPIs) have reduced the circulation of common respiratory pathogens such as influenza, leading to a marked decrease in mortality caused by respiratory diseases in January-February 2021, a period that normally sees peak winter infections. Because respiratory infections can precipitate deaths from other conditions, their absence likely also reduced mortality from causes that depend on them, especially in adults aged 80 and over. This mechanism may account for the mental- and neurological-disease-related death deficits observed in early 2021 among the 65–79 and 80 + age groups. On the other hand, the implementation of NPIs has also been used to explain the slight decrease in mortality attributed to cancer during the COVID-19 due to delays in diagnosis observed in the UK [3]. However, this effect is unlikely in Switzerland, where oncology care remained accessible and control measures remained comparatively less stringent [26].

### Cross-cause correlation

Our multivariate model revealed two layers of pre-2020 cause-specific mortality correlation: first, most causes share a winter peak across age groups; second, even after removing this seasonal component, excess deaths from respiratory, cardiovascular, and mental-neurological diseases remain tightly coupled. Modelling these correlations jointly made it possible to distinguish between competing explanations for cause-specific mortality patterns (i.e., misclassification, mortality displacement, and indirect effects) which cannot be disentangled when causes are analysed in isolation. The correlation was highest in winters with the largest influenza epidemics, implying that a substantial share of the apparent cardiovascular excess was infection-related (either through biologically triggered events or misclassification) [27, 28]. While extreme weather or pollution could still contribute, previous studies have suggested that they only explain a minor fraction of the observed excess mortality [29, 30]. Evidence from 2020 to 21 reinforces this assumption, as excess cardiovascular and mental/neurological deaths correlate with both COVID-19 and non-COVID-19 respiratory excesses during a period when mortality patterns were disrupted. In this context, the COVID-19 disruption served as a natural experiment, as COVID-19 waves occurred at unusual periods for a respiratory disease and flu waves were almost non-existent in winter 2020–21. While the exact mechanisms need to be investigated in more depth, this suggests that the contribution of respiratory pathogens on overall mortality has been underestimated.

### Strengths, limitations and perspectives

The study has several strengths. We studied the COVID-19 disruption, which temporarily decoupled normally synchronized causes of death, to examine how they are linked. We built a multi-dimensional Bayesian model that captured population change, long-term trends, seasonal patterns, and cross-cause correlation. Gaussian processes gave this model more flexibility than generalized linear models with splines or seasonal ARIMA, resulting in sharper estimates and more realistic uncertainty [4], [9–12], [31]. Other approaches that have formally modeled cross-cause correlation, used tensors or multi-cause Gaussian processes, but their complexity often prevents a clear understanding of how cause-specific mortalities are related [8, 32]. Our Bayesian framework explicitly propagated uncertainty, a critical feature when excess-mortality estimates depend on counterfactual predictions. The analysis also benefited from Switzerland’s high-quality death-certificate data, which follow consistent coding guidelines and achieve near-complete coverage. Weekly aggregation preserved high temporal granularity and allowed us to detect fine-scale synchrony between causes.

The study also has limitations. First, most analyses rely solely on the unique underlying cause of death, which may overlook mechanisms such as mortality displacement or competing risks. Switzerland’s death certificates record immediate, underlying, and concomitant causes, and future work should leverage this richer information to move beyond the underlying cause of death. Incorporating secondary and contributing causes would allow a more rigorous quantification of mortality displacement across time and causes, and would help disentangle misclassification from true shifts in cause-specific mortality. This was illustrated in our preliminary analysis focusing on cancer and more comprehensively in Duràn et al. [17], and could be further extended using more formal modelling approaches. Frailty-survival models capture temporal displacement by accounting for unobserved heterogeneity, while competing-risk models capture cross-cause displacement by redistributing deaths among alternatives [5, 33]. Misclassification, however, may persist even with richer cause-of-death information. Second, some model assumptions, such as the static effects across sex, region, and broad age ranges may oversimplify reality. It also applies to the estimation of excess mortality in the other causes category, which, by definition, comprises a highly heterogeneous set of conditions. For this reason, we did not present or interpret these results, and the magnitude of the corresponding all-cause excess mortality estimates (Figure 3) should be interpreted with caution. Third, reliance on aggregated data raises the risk of ecological bias: correlations seen at the population level, e.g., between COVID-19 and cardiovascular mortality, do not imply causation for individual cases. Finally, the dataset ends in 2021, limiting our ability to assess the long-term effects of COVID-19 on cause-specific mortality.

In the future, cause-specific mortality data could serve different purposes. First, it shows how disruption in one cause, such as a respiratory virus epidemic, can cascade into shifts in other mortality patterns, revealing hidden dependencies. Second, incorporating these dependencies to predict all-cause mortality might improve accuracy. Third, such data could be used to explain the divergences in excess mortality patterns observed between countries during the COVID-19 pandemic. More immediately, several extensions are feasible. We could apply the same framework to other countries to test whether the correlation patterns we observed in Switzerland are generalisable. Further, stratifying the analysis by socio-economic status would allow us to characterize the mortality burden across different levels of vulnerability. Finally, extending the series through 2022 and 2023 will clarify whether the different effects responsible for the disruption of mortality persist after 2021.

### Conclusion

The COVID-19 pandemic caused an exceptional disruption in mortality patterns, affecting multiple causes of death simultaneously. This disruption provided a unique natural experiment to disentangle mechanisms that have historically overlapped, including misclassification, mortality displacement across time and causes, and the effects of control measures. Our analysis offers a first step toward understanding the links between causes of death that normally evolve in parallel. The next step will be to integrate multiple-cause-of-death data and explicitly model complex processes such as the harvesting effect and competing risks to achieve a more complete representation of mortality dynamics.
