# Supplementary material for: COVID-19 disrupted patterns of cause-specific mortality in Switzerland
Source: Int J Public Health. 2026 Jun 23;71:1609856. doi: 10.3389/ijph.2026.1609856 (PMC13338603; doi:10.3389/ijph.2026.1609856)
Supplement: Supplementary file 1 [file Presentation1.pdf]

Supplementary File 1: COVID-19 disrupted patterns of cause-specific mortality in Switzerland

1 Table

Table S1. Description of the nine categories used to group the causes of deaths. Chapters correspond to the International Classification of Diseases, 10th Revision (ICD-10).

Cause	Examples	Chapters
Cardiovascular Diseases	Ischaemic heart disease, heart attack	9
Neoplasms (Cancers)	Lung, breast, prostate neoplasms	2
Mental and Neurological Disorders	Dementia, Alzheimer, Parkinson	5, 6
Respiratory Diseases	Pulmonary disease, pneumonia, influenza	10
External Causes	Fall, vehicule accident	20
Suicide	Intentional self-harm/poisoning	20
Infectious and Parasitic Diseases	Sepsis	1
Other Causes	Unspecified, diabetes, senility, organ disease	3, 4, 7, 8, 11, 12, 13, 14, 15, 16, 17, 18, 22
COVID-19	COVID-19	22

2 Figures

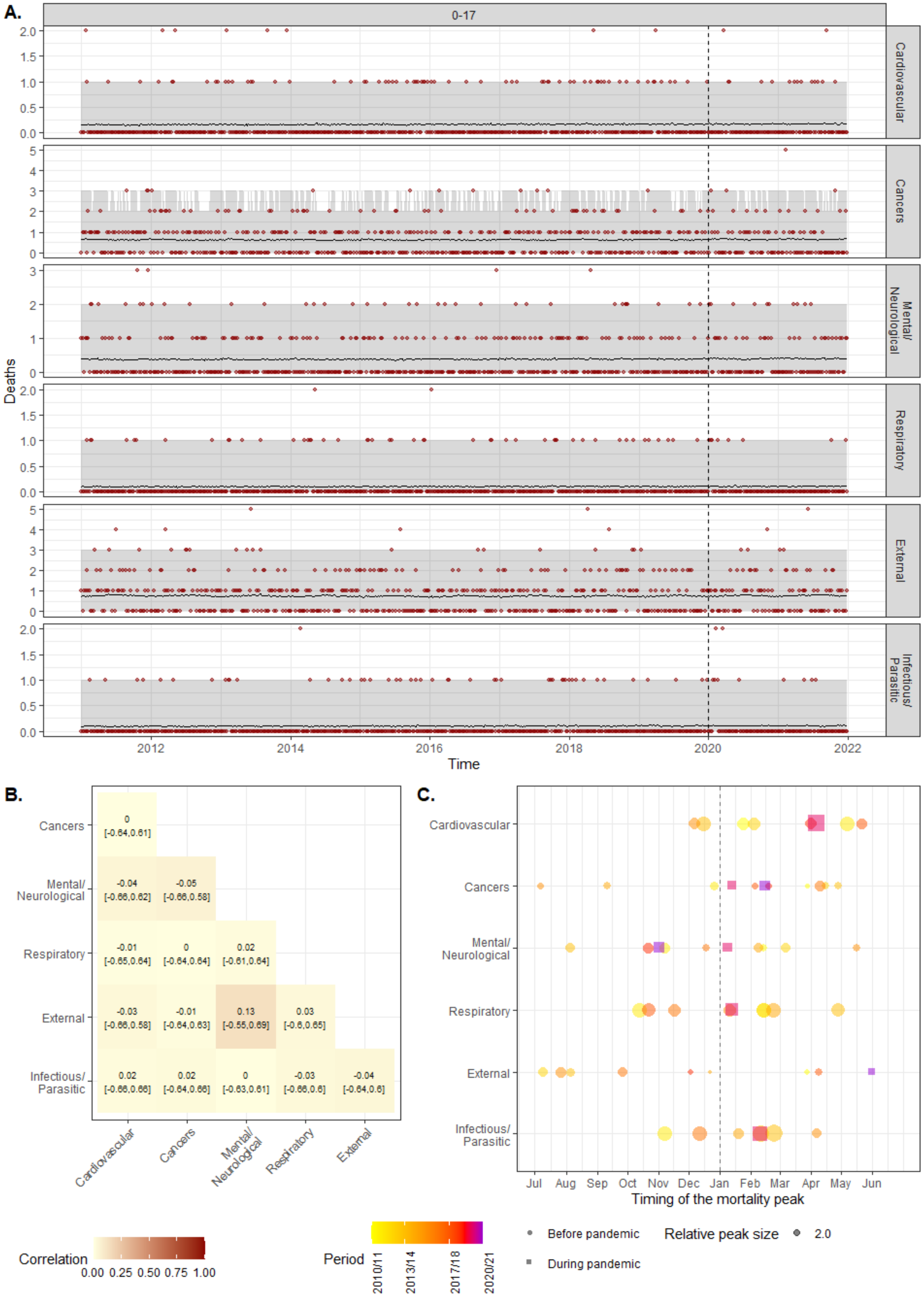
A.
0-17
Cardiovascular
Cancers
Mental/
Neurological
Respiratory
External
Infectious/
Parasitic
Deaths
2012
2014
2016
2018
2020
2022
Time
B.
Cancers
Mental/
Neurological
Respiratory
External
Infectious/
Parasitic
0
[-0.64,0.61]
-0.04
[-0.66,0.62]
-0.05
[-0.66,0.58]
-0.01
[-0.65,0.64]
0
[-0.64,0.64]
0.02
[-0.61,0.64]
-0.03
[-0.66,0.58]
-0.01
[-0.64,0.63]
0.13
[-0.55,0.69]
0.03
[-0.6,0.65]
0.02
[-0.66,0.66]
0.02
[-0.64,0.66]
0
[-0.63,0.61]
-0.03
[-0.66,0.6]
-0.04
[-0.64,0.6]
Cardiovascular
Cancers
Mental/
Neurological
Respiratory
External
C.
Cardiovascular
Cancers
Mental/
Neurological
Respiratory
External
Infectious/
Parasitic
Jul
Aug
Sep
Oct
Nov
Dec
Jan
Feb
Mar
Apr
May
Jun
Timing of the mortality peak
Correlation
0.00 0.25 0.50 0.75 1.00
Period
2010/11
2013/14
2017/18
2020/21
Before pandemic
During pandemic
Relative peak size
2.0

Figure S1. Model fit and cross-cause dependencies for the 0-17 age group. A. Model fit. Number of weekly deaths observed in 2011-21 (red points) and model estimates (before 2020) and predictions (2020-21) (black line) with 95% credibility intervals (gray area), for the six main causes of deaths for the 80+ age group. B. Cross-cause correlation matrix of the excess mortality for 0-17 age group. C. Timing of the mortality peak. The color points and squares correspond to observed peak for each winter (we considered the period from July to June of the next year as peak usually occured during winter). The black points and lines represent the posterior estimate of the peak with 95% credibility intervals. Posterior estimates are only reported when the credible arc length is below 180 days, as larger values indicate weak seasonal concentration and indicate that no clear peak can be identified from the data.

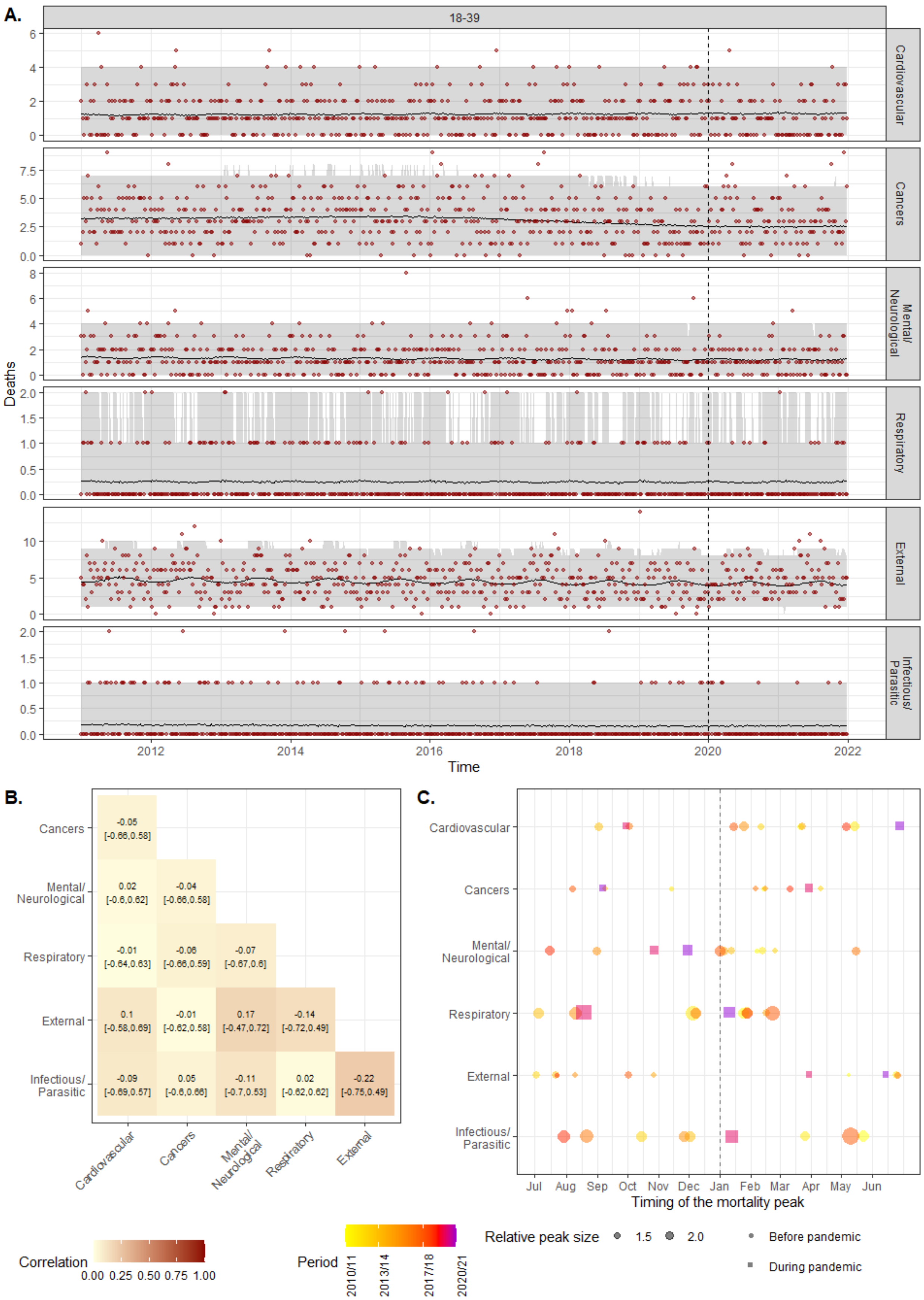

A.
18-39
Cardiovascular
Cancers
Mental/ Neurological
Respiratory
External
Infectious/ Parasitic
Deaths
Time
2012
2014
2016
2018
2020
2022
B.
Cancers
Mental/ Neurological
Respiratory
External
Infectious/ Parasitic
Cardiovascular
Cancers
Mental/ Neurological
Respiratory
External
-0.05 [-0.66,0.58]
0.02 [-0.6,0.62]
-0.04 [-0.66,0.58]
-0.01 [-0.64,0.63]
-0.06 [-0.66,0.59]
-0.07 [-0.67,0.6]
0.1 [-0.58,0.69]
-0.01 [-0.62,0.58]
0.17 [-0.47,0.72]
-0.14 [-0.72,0.49]
-0.09 [-0.69,0.57]
0.05 [-0.6,0.66]
-0.11 [-0.7,0.53]
0.02 [-0.62,0.62]
-0.22 [-0.75,0.49]
C.
Cardiovascular
Cancers
Mental/ Neurological
Respiratory
External
Infectious/ Parasitic
Jul
Aug
Sep
Oct
Nov
Dec
Jan
Feb
Mar
Apr
May
Jun
Timing of the mortality peak
Correlation
0.00 0.25 0.50 0.75 1.00
Period
2010/11
2013/14
2017/18
2020/21
Relative peak size
1.5
2.0
Before pandemic
During pandemic

Figure S2. Model fit and cross-cause dependencies for the 18-39 age group. A. Model fit. Number of weekly deaths observed in 2011-21 (red points) and model estimates (before 2020) and predictions (2020-21) (black line) with 95% credibility intervals (gray area), for the six main causes of deaths for the 80+ age group. B. Cross-cause correlation matrix of the excess mortality for 18-39 age group. C. Timing of the mortality peak. The color points and squares correspond to observed peak for each winter (we considered the period from July to June of the next year as peak usually occured during winter). The black points and lines represent the posterior estimate of the peak with 95% credibility intervals. Posterior estimates are only reported when the credible arc length is below 180 days, as larger values indicate weak seasonal concentration and indicate that no clear peak can be identified from the data.

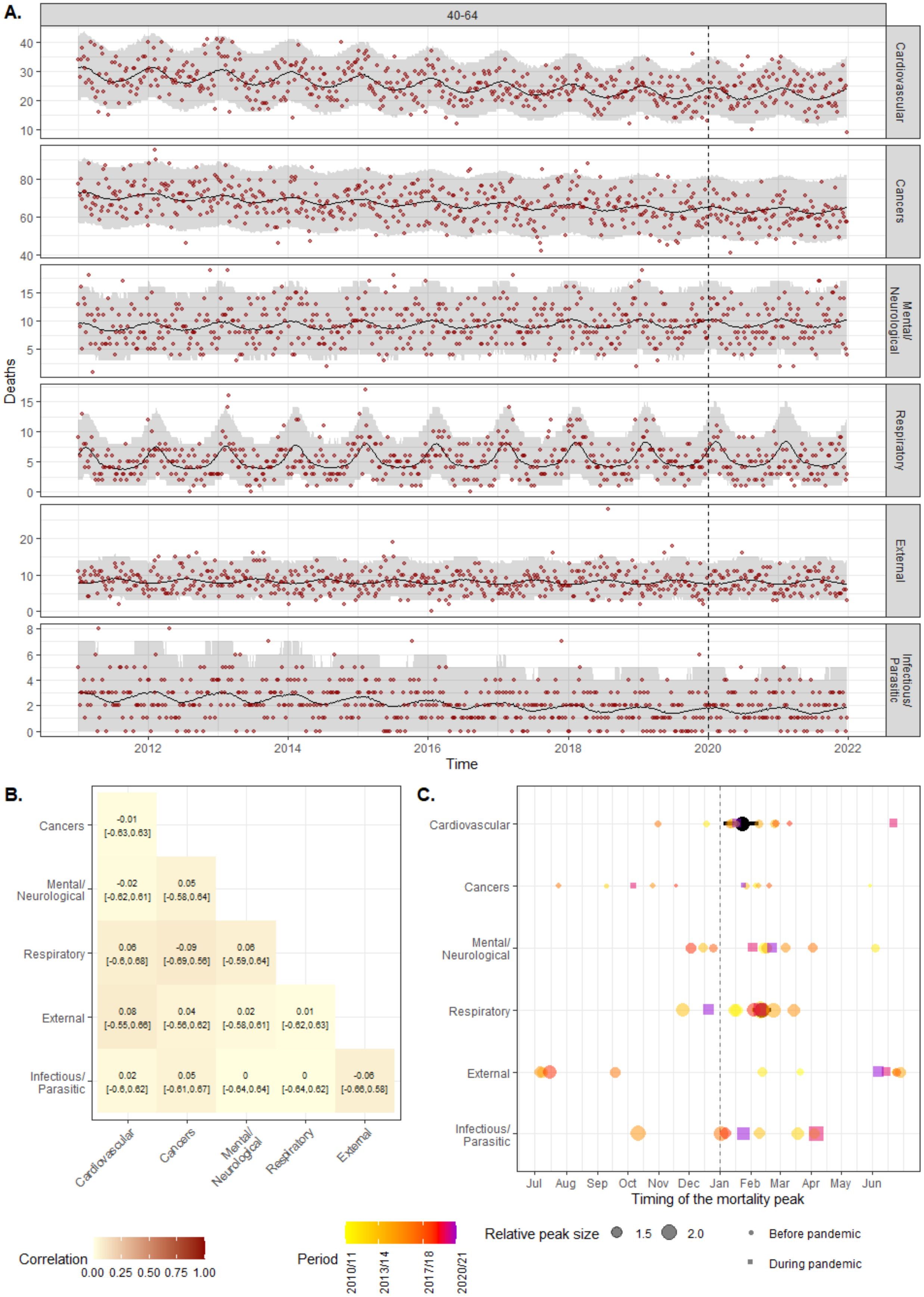
A.
40-64
Cardiovascular
Cancers
Mental/
Neurological
Respiratory
External
Infectious/
Parasitic
Deaths
Time
2012
2014
2016
2018
2020
2022
B.
Cancers
Mental/
Neurological
Respiratory
External
Infectious/
Parasitic
-0.01
[-0.63,0.63]
-0.02
[-0.62,0.61]
0.05
[-0.58,0.64]
0.06
[-0.6,0.68]
-0.09
[-0.69,0.56]
0.06
[-0.59,0.64]
0.08
[-0.55,0.66]
0.04
[-0.56,0.62]
0.02
[-0.58,0.61]
0.01
[-0.62,0.63]
0.02
[-0.6,0.62]
0.05
[-0.61,0.67]
0
[-0.64,0.64]
0
[-0.64,0.62]
-0.06
[-0.66,0.58]
Cardiovascular
Cancers
Mental/
Neurological
Respiratory
External
C.
Cardiovascular
Cancers
Mental/
Neurological
Respiratory
External
Infectious/
Parasitic
Jul
Aug
Sep
Oct
Nov
Dec
Jan
Feb
Mar
Apr
May
Jun
Timing of the mortality peak
Correlation
0.00 0.25 0.50 0.75 1.00
Period
2010/11
2013/14
2017/18
2020/21
Relative peak size
1.5
2.0
Before pandemic
During pandemic

Figure S3. Model fit and cross-cause dependencies for the 40-64 age group. A. Model fit. Number of weekly deaths observed in 2011-21 (red points) and model estimates (before 2020) and predictions (2020-21) (black line) with 95% credibility intervals (gray area), for the six main causes of deaths for the 80+ age group. B. Cross-cause correlation matrix of the excess mortality for 40-64 age group. C. Timing of the mortality peak. The color points and squares correspond to observed peak for each winter (we considered the period from July to June of the next year as peak usually occured during winter). The black points and lines represent the posterior estimate of the peak with 95% credibility intervals. Posterior estimates are only reported when the credible arc length is below 180 days, as larger values indicate weak seasonal concentration and indicate that no clear peak can be identified from the data.

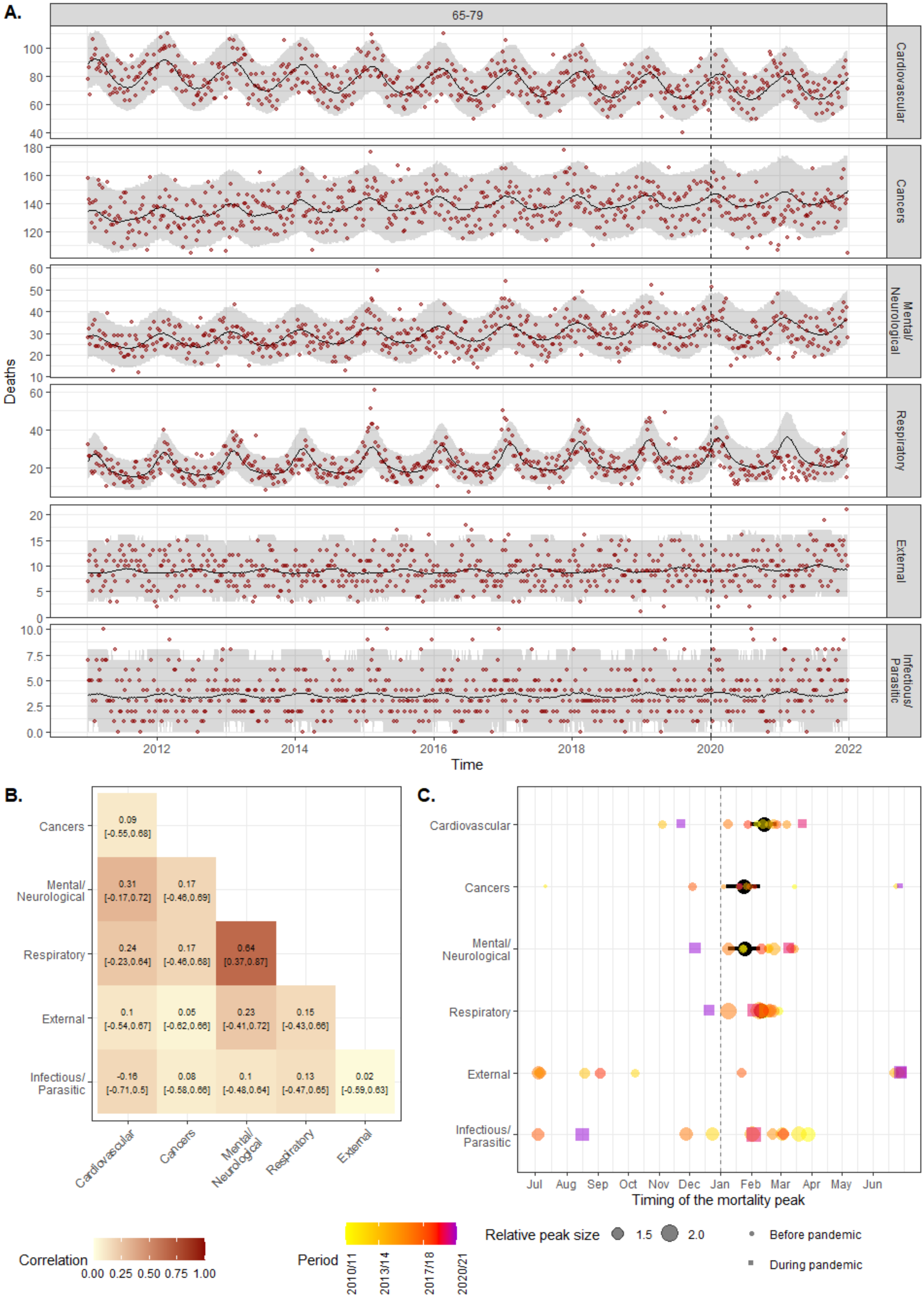
A.
65-79
Cardiovascular
Cancers
Mental/
Neurological
Respiratory
External
Infectious/
Parasitic
Deaths
Time
2012
2014
2016
2018
2020
2022
B.
Cancers
Mental/
Neurological
Respiratory
External
Infectious/
Parasitic
0.09
[-0.55,0.68]
0.31
[-0.17,0.72]
0.17
[-0.46,0.69]
0.24
[-0.23,0.64]
0.17
[-0.46,0.68]
0.64
[0.37,0.87]
0.1
[-0.54,0.67]
0.05
[-0.62,0.66]
0.23
[-0.41,0.72]
0.15
[-0.43,0.66]
-0.16
[-0.71,0.5]
0.08
[-0.58,0.66]
0.1
[-0.48,0.64]
0.13
[-0.47,0.65]
0.02
[-0.59,0.63]
Cardiovascular
Cancers
Mental/
Neurological
Respiratory
External
C.
Cardiovascular
Cancers
Mental/
Neurological
Respiratory
External
Infectious/
Parasitic
Jul
Aug
Sep
Oct
Nov
Dec
Jan
Feb
Mar
Apr
May
Jun
Timing of the mortality peak
Correlation
0.00
0.25
0.50
0.75
1.00
Period
2010/11
2013/14
2017/18
2020/21
Relative peak size
1.5
2.0
Before pandemic
During pandemic

Figure S4. Model fit and cross-cause dependencies for the 65-79 age group. A. Model fit. Number of weekly deaths observed in 2011-21 (red points) and model estimates (before 2020) and predictions (2020-21) (black line) with 95% credibility intervals (gray area), for the six main causes of deaths for the 80+ age group. B. Cross-cause correlation matrix of the excess mortality for 65-79 age group. C. Timing of the mortality peak. The color points and squares correspond to observed peak for each winter (we considered the period from July to June of the next year as peak usually occured during winter). The black points and lines represent the posterior estimate of the peak with 95% credibility intervals. Posterior estimates are only reported when the credible arc length is below 180 days, as larger values indicate weak seasonal concentration and indicate that no clear peak can be identified from the data.

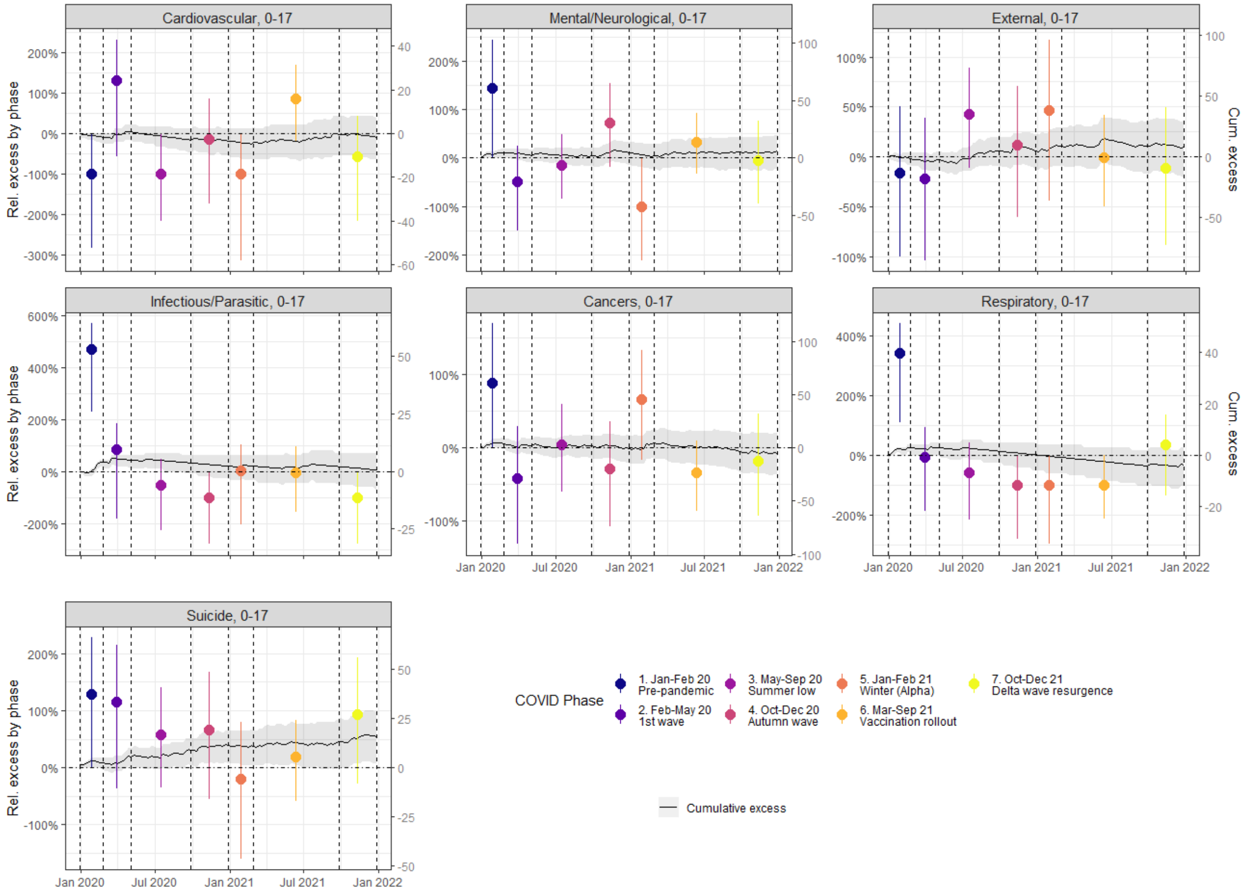

Figure S5. Cause-specific excess mortality by phase for 0-17 age group. Black lines and gray area correspond to posterior mean and 95% credibility intervals of the cumulative (relative and absolute) excess mortality over 2020-21, respectively. The colored points and vertical lines corresponds to posterior mean and 95% credibility intervals of the relative excess mortality by phase, respectively. The phases are delimited by the dashed vertical lines.

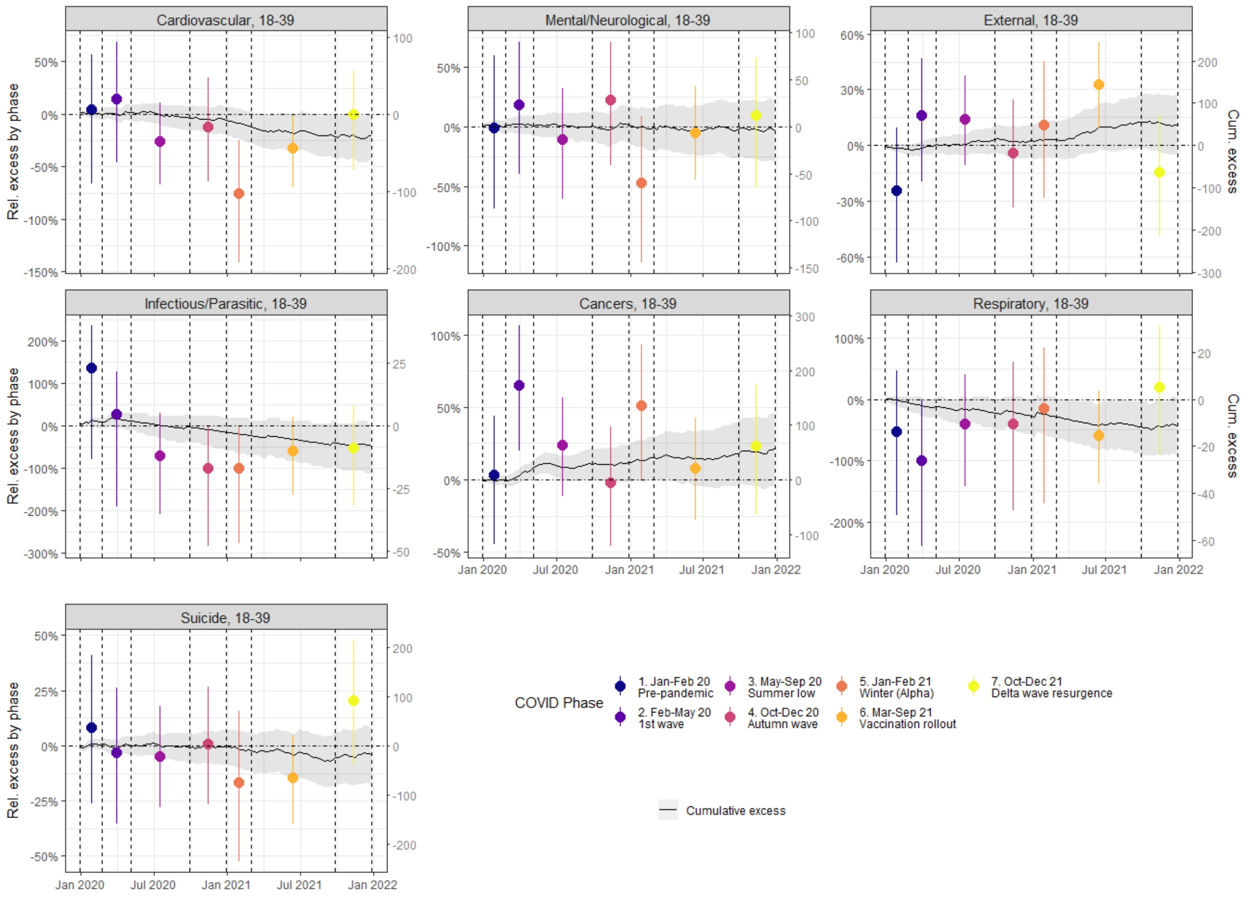

Figure S6. Cause-specific excess mortality by phase for 18-39 age group. Black lines and gray area correspond to posterior mean and 95% credibility intervals of the cumulative (relative and absolute) excess mortality over 2020-21, respectively. The colored points and vertical lines corresponds to posterior mean and 95% credibility intervals of the relative excess mortality by phase, respectively. The phases are delimited by the dashed vertical lines.

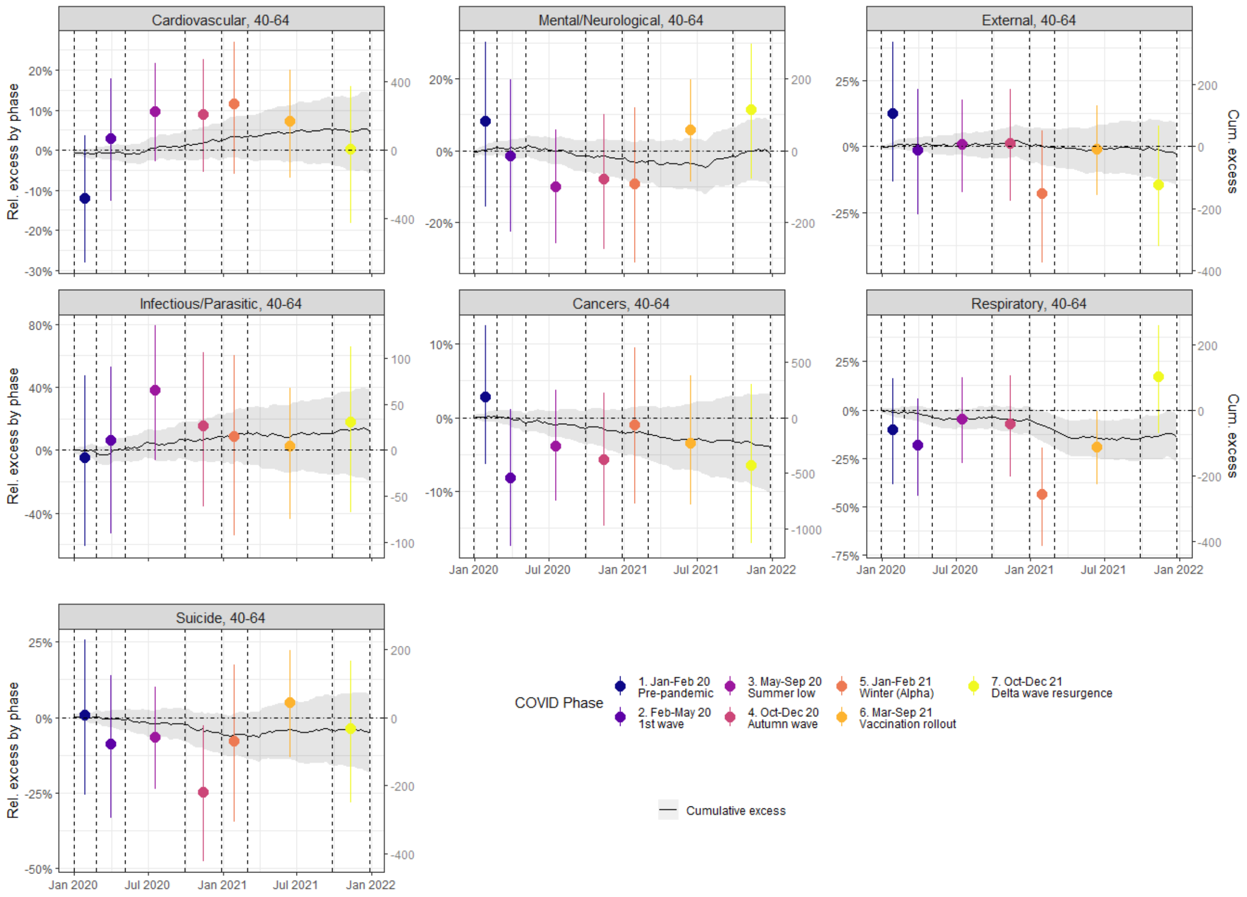

Figure S7. Cause-specific excess mortality by phase for 40-64 age group. Black lines and gray area correspond to posterior mean and 95% credibility intervals of the cumulative (relative and absolute) excess mortality over 2020-21, respectively. The colored points and vertical lines corresponds to posterior mean and 95% credibility intervals of the relative excess mortality by phase, respectively. The phases are delimited by the dashed vertical lines.

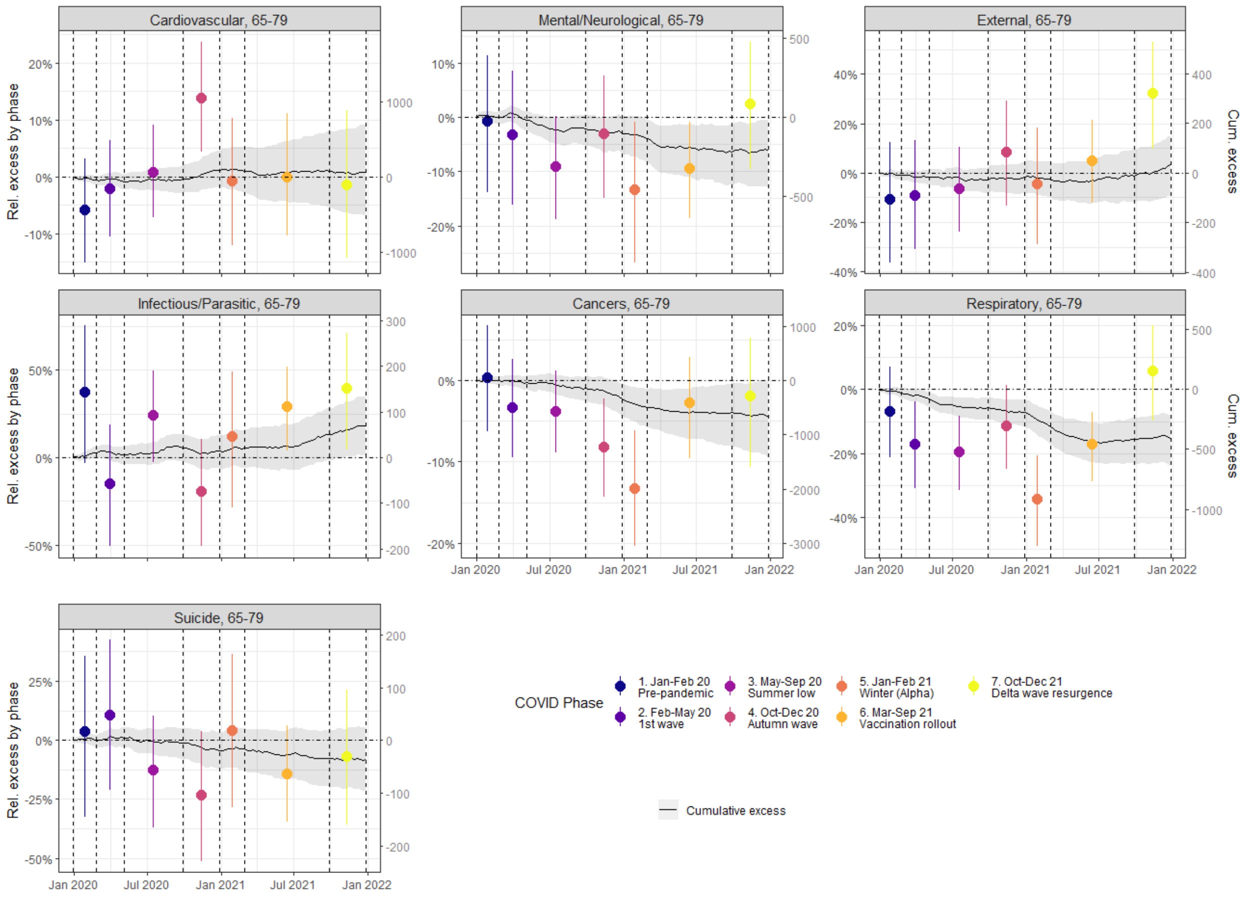

Figure S8. Cause-specific excess mortality by phase for 65-79 age group. Black lines and gray area correspond to posterior mean and 95% credibility intervals of the cumulative (relative and absolute) excess mortality over 2020-21, respectively. The colored points and vertical lines corresponds to posterior mean and 95% credibility intervals of the relative excess mortality by phase, respectively. The phases are delimited by the dashed vertical lines.

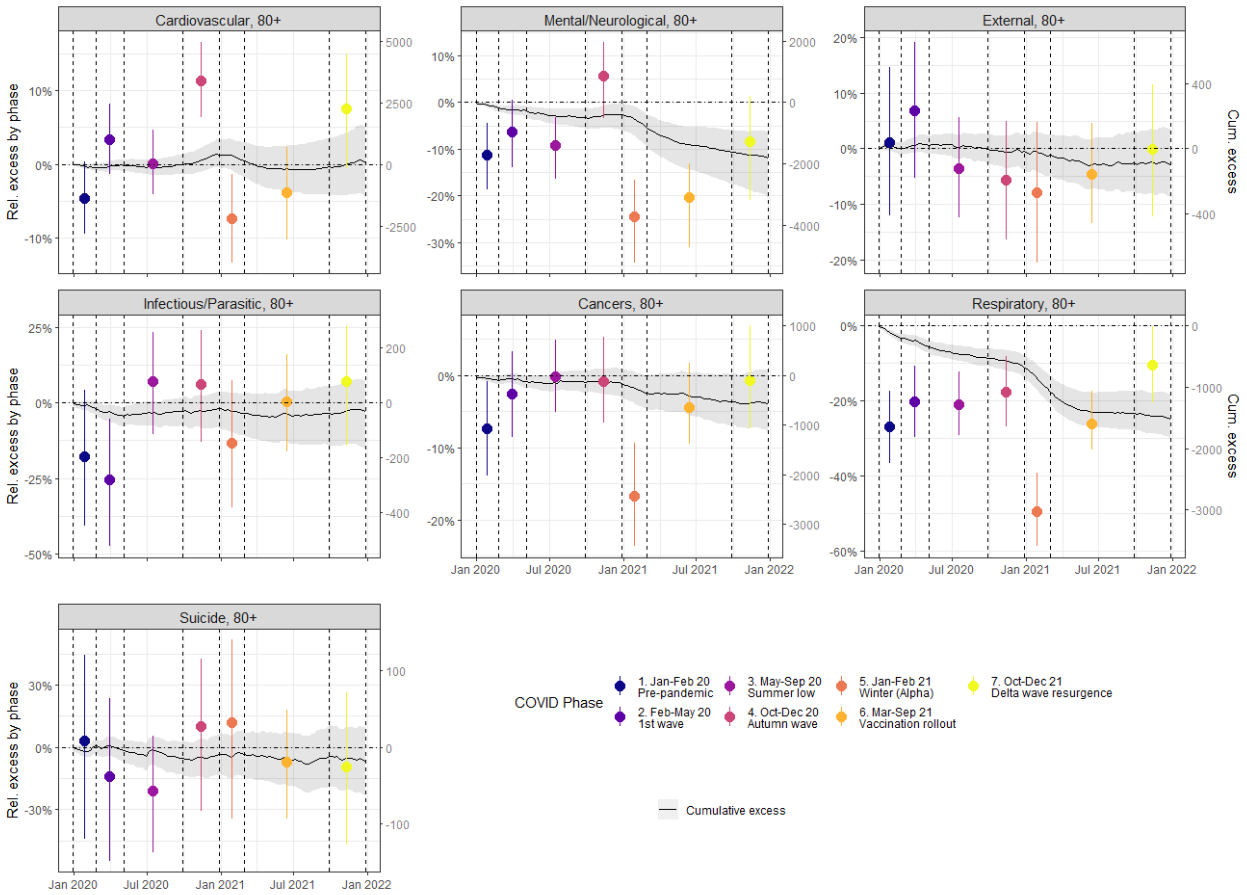

Figure S9. Cause-specific excess mortality by phase for 80+ age group. Black lines and gray area correspond to posterior mean and 95% credibility intervals of the cumulative (relative and absolute) excess mortality over 2020-21, respectively. The colored points and vertical lines corresponds to posterior mean and 95% credibility intervals of the relative excess mortality by phase, respectively. The phases are delimited by the dashed vertical lines.

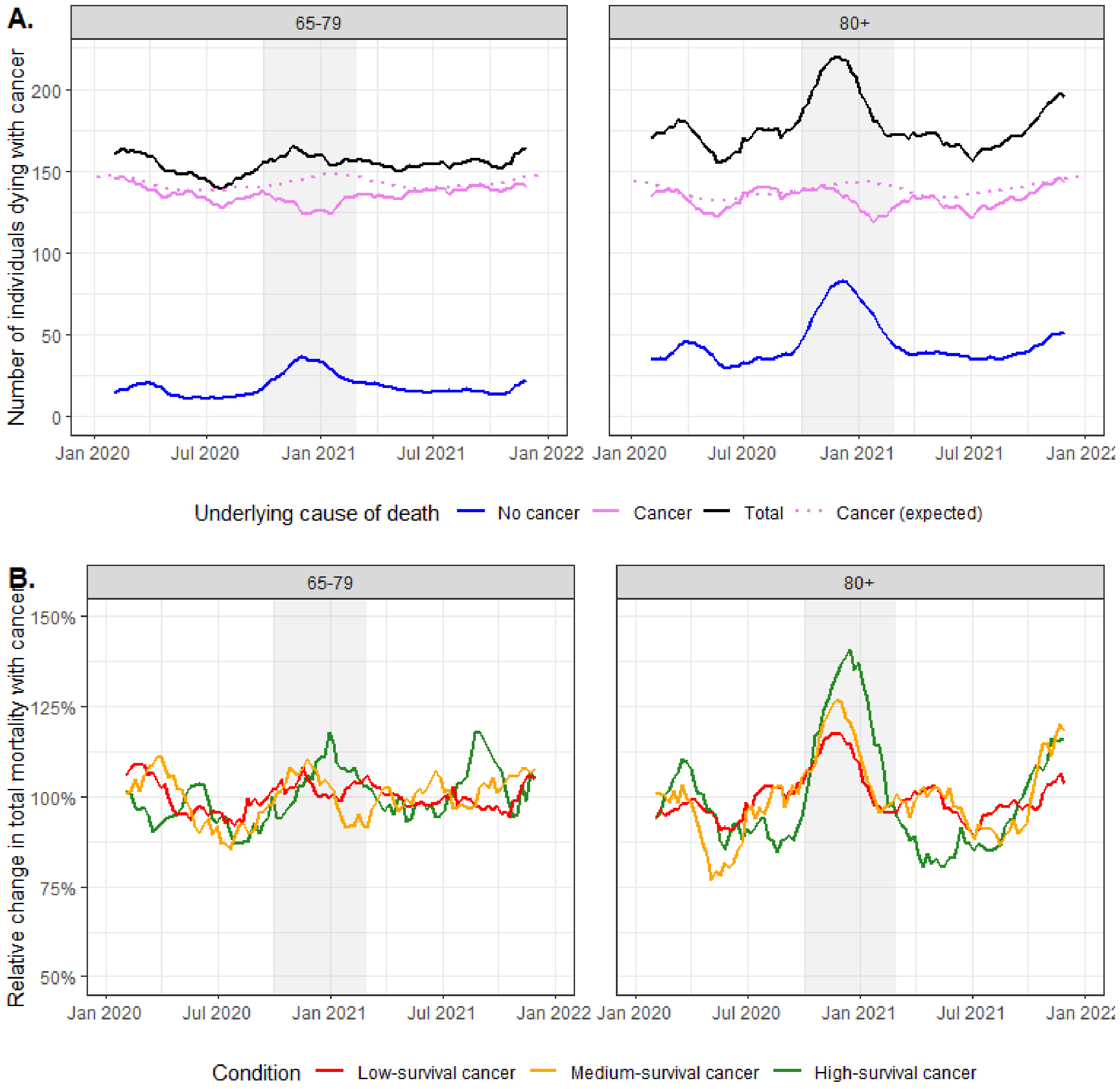

Figure S10. Additional analysis of cancer mortality during COVID-19 pandemic. A. Number of individuals dying with cancer over time in 2020-21, stratified by the underlying cause of deaths and age groups (65-79 and 80+). The pink lines represent the number of deaths from cancer (i.e., whose underlying cause is cancer), either observed (straight lines) or expected (dashed lines), the blue lines represent the number individuals with cancer dying from other causes and the black lines represent the total number of individuals dying with cancer. The gray area denotes the period where a deficit in cancer mortality was observed. For better visualisation, a moving average of 9 weeks was applied to the weekly numbers. B. Relative change in total number of individuals dying with cancer, stratified by cancer survival (low, medium and high) and age groups (65-79 and 80+). The change was calculated relative to the mean number of deaths during 2020-21 for each age group and survival group. For ages 65–79, cancers with high survival included breast, male genital, thyroid/endocrine, skin (melanoma), and benign or in situ neoplasms; medium survival included digestive, urinary, female genital, oral/pharyngeal, lymphoid/haematopoietic (except myeloma and

leukaemias), and uncertain/unknown neoplasms; low survival included respiratory, mesothelial/soft tissue, CNS, bone, and ill-defined cancers. For ages 80+, high survival included breast, male genital, benign, and in situ neoplasms; medium survival included colorectal, thyroid/endocrine, urinary, skin (melanoma), and uncertain/unknown neoplasms; and low survival included respiratory, other digestive (e.g., oesophagus, stomach, pancreas, liver), lymphoid/haematopoietic, female genital, mesothelial/soft tissue, CNS, bone, and ill-defined cancers.

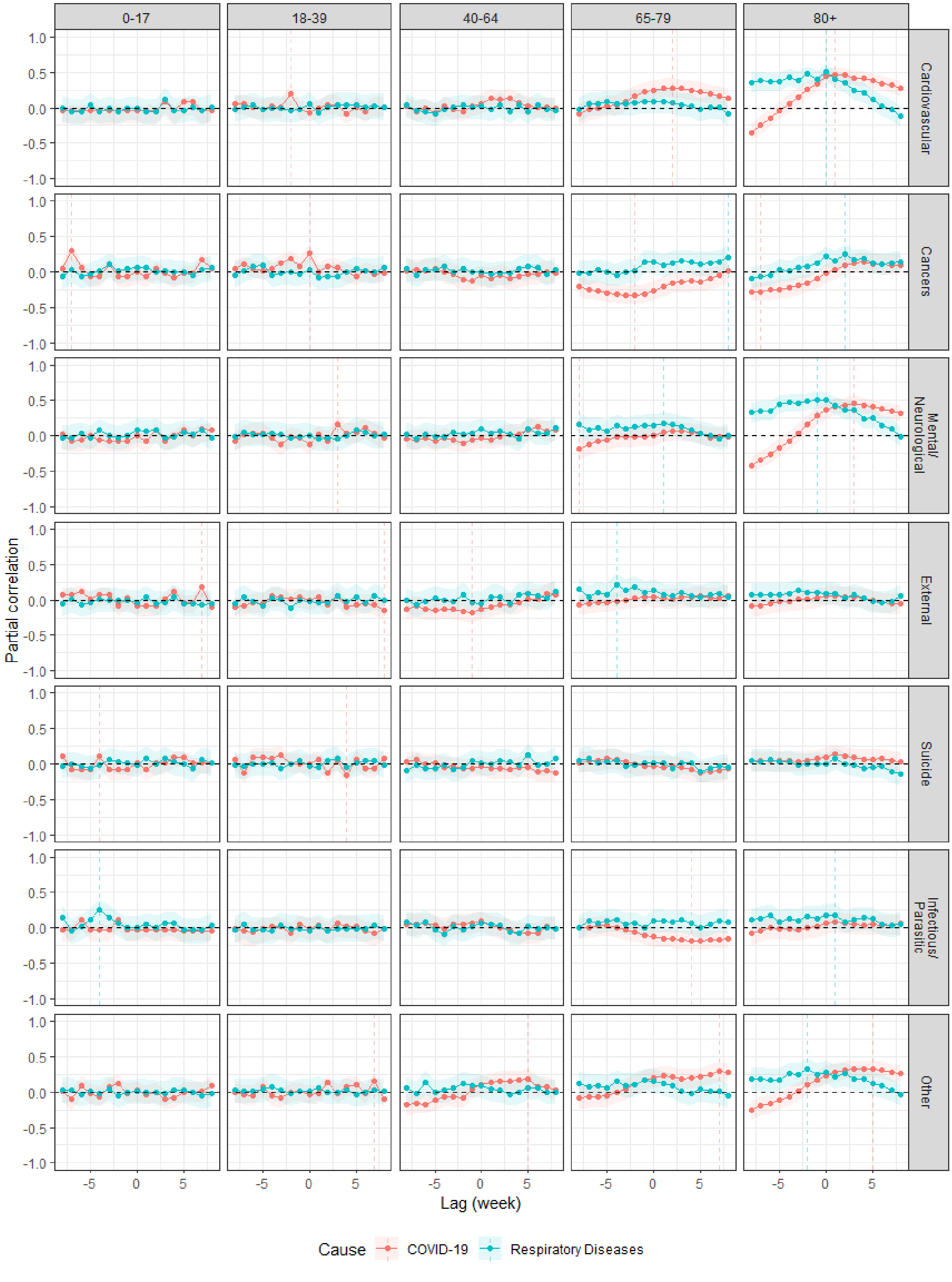

0-17
18-39
40-64
65-79
80+
Cardiovascular
Cancers
Mental/ Neurological
External
Suicide
Infectious/ Parasitic
Other
Partial correlation
1.0
0.5
0.0
-0.5
-1.0
-5
0
5
Lag (week)
Cause
COVID-19
Respiratory Diseases

Figure S11. Partial correlation between excess mortality due to either COVID-19 (red) or to other respiratory diseases (blue, tagged as "Respiratory Diseases") and excess mortality due to specific causes of deaths for each age group, considering different lags (in weeks), i.e. different time shifts between changes in excess mortality due to respiratory diseases and changes in excess mortality due to the considered cause. Solid color lines correspond to the posterior mean and shaded colored area to the 95% credibility intervals. The vertical dashed lines indicate the lag with the highest absolute partial correlation. A positive lag corresponds to the situation where either excess mortality due to respiratory diseases (either COVID-19 or other respiratory diseases) occurs slightly after excess mortality due to considered cause.
